# Immunopathogenesis, Diagnosis, and Treatment of Hashimoto’s Thyroiditis

**DOI:** 10.26502/aimr.0232

**Published:** 2026-01-07

**Authors:** Felicia Delgadillo, Devendra K. Agrawal

**Affiliations:** 1 Department of Translational Research, College of Osteopathic Medicine of the Pacific, Western University of Health Sciences, Pomona, California 91766 USA

**Keywords:** Autoimmunity, Autoimmune thyroid disorder, Hashimoto’s thyroiditis, HLA typing, Immune cells, Immunological tolerance, Oxidative stress, Selenium, Susceptibility genes, Th17 cells, Thyrocyte destruction, Thyroglobulin, Thyroid peroxides, Vitamin D

## Abstract

This comprehensive literature review examines the immunopathogenesis of Hashimoto’s thyroiditis, a prevalent autoimmune thyroid disorder. Hashimoto’s thyroiditis results from a complex interplay of genetic predisposition and environmental triggers, leading to loss of immune tolerance to thyroid antigens. Hashimoto’s thyroiditis involves both cellular (T cells) and humoral (B cells, autoantibodies) responses. Key players include Th1, Th17, and regulatory T cells, with an imbalance in the Th17/Treg ratio implicated. The diagnosis of Hashimoto’s thyroiditis relies on clinical presentation, thyroid function tests, detection of anti-thyroid peroxidase and anti-thyroglobulin antibodies, and ultrasound imaging. Current treatment of Hashimoto’s thyroiditis primarily involves levothyroxine replacement therapy. Emerging adjunctive approaches include vitamin D and selenium supplementation. Some potential challenges of Hashimoto’s thyroiditis include understanding precise disease triggers, developing predictive biomarkers, and addressing persistent symptoms despite biochemical euthyroid. This article highlights recent advances in understanding the pathogenesis of Hashimoto’s thyroiditis, particularly the roles of Th17 cells and oxidative stress. It also discusses controversies and knowledge gaps, such as the exact mechanisms that initiate autoimmunity and the factors that influence disease progression. There is a need for personalized treatment strategies and therapies targeting the underlying autoimmune process for better treatment of patients with Hashimoto’s Thyroiditis.

## 1. Introduction

Hashimoto’s thyroiditis (HT), also known as chronic lymphocytic thyroiditis, constitutes the predominant form of autoimmune thyroid disease (AITD) and represents a leading cause of hypothyroidism in regions with adequate iodine intake [1,2]. First documented by Hakaru Hashimoto in 1912 as *struma lymphomatosa*, its characterization initiated the understanding of autoimmune destruction as a disease mechanism [2–4]. The disease manifests through a complex interplay of genetic predispositions and environmental factors, culminating in lymphocytic infiltration of the thyroid gland, cellular damage, and ultimately, impaired thyroid function [1,2]. A defining immunological feature of HT is the presence of circulating autoantibodies against thyroid peroxidase (TPOAb) and thyroglobulin (TgAb) [2,5]. The chronic inflammatory process within the thyroid often leads to glandular fibrosis and atrophy, necessitating lifelong thyroid hormone replacement therapy in many affected individuals [1,5]. The prevalence of HT continues to rise, impacting a substantial segment of the global population [6]. Its chronic nature and potential progression to overt hypothyroidism necessitate continuous management and can significantly affect patients’ quality of life [1]. Beyond thyroid dysfunction, HT is frequently associated with other autoimmune conditions, underscoring its systemic implications within the broader landscape of autoimmunity [1,3,6,7]. Despite extensive research, the precise triggers and intricate molecular mechanisms that initiate thyroid destruction in HT remain partially understood [3,5,8]. A comprehensive synthesis of current knowledge regarding the immunological processes underlying HT is therefore warranted to consolidate fragmented findings, identify persistent knowledge gaps, and guide future investigative priorities.

### 1.1 Background

The conceptualization of autoimmune thyroid disease, and particularly Hashimoto’s thyroiditis, has evolved significantly since Hakaru Hashimoto’s initial description in 1912 [9,10]. Hashimoto’s pioneering work elucidated the distinct histological features of lymphocytic infiltration, follicular cell degeneration, and fibrosis that differentiated this condition from other forms of goiter prevalent at the time [6,10]. A pivotal development occurred decades later, beginning with Noel Rose’s studies in the mid-20th century. Rose’s experiments, involving the injection of thyroglobulin back into the same animal from which it was derived, demonstrated the production of autoantibodies and inflammation within the thyroid, thereby leading to the discovery of autoimmune thyroiditis [3]. This seminal finding was swiftly followed by Doniach and Roitt’s detection of thyroglobulin antibodies in the serum of HT patients, providing the first serological evidence for the autoimmune nature of the disease [3,10]. Subsequent investigations by Rose and others expanded this understanding, identifying the influence of both major histocompatibility complex (MHC) and non-MHC genes, the role of environmental factors, and the presence of autoreactive T cells even in healthy individuals, underscoring the necessity of active suppressive mechanisms to avert autoimmunity [3]. These historical milestones established the foundation for contemporary research into the complex immunopathology of HT.

Autoimmunity typically involves a breakdown in immunological tolerance, resulting in an immune response directed against self-antigens [3,5]. This loss of tolerance can arise from defects in central tolerance mechanisms, where self-reactive lymphocytes are typically eliminated during development, or from disturbances in peripheral tolerance, which controls autoreactive cells that escape central deletion [5]. In HT, immune cells, particularly T and B lymphocytes, aberrantly recognize thyroid-specific antigens, initiating a destructive inflammatory cascade [5,11]. The process involves the activation of antigen-presenting cells (APCs), such as dendritic cells, which present thyroid autoantigens to naive CD4+ T cells [1,5]. This activation leads to the differentiation and clonal expansion of autoreactive T cells, followed by the activation of subsequent B cells, culminating in the production of autoantibodies and the progressive destruction of the thyroid [1,5,12]. Both cellular responses, mediated by cytotoxic T cells, and humoral responses, involving antibody production, contribute to the characteristic immune attack observed in HT [5]. Imbalances in T helper cell subsets, particularly an intensified Th1 and Th17 response, coupled with deficiencies in regulatory T cells (Tregs), are often implicated in exacerbating the inflammatory environment and disrupting immune homeostasis [1,5,13,14].

**Figure 1:**
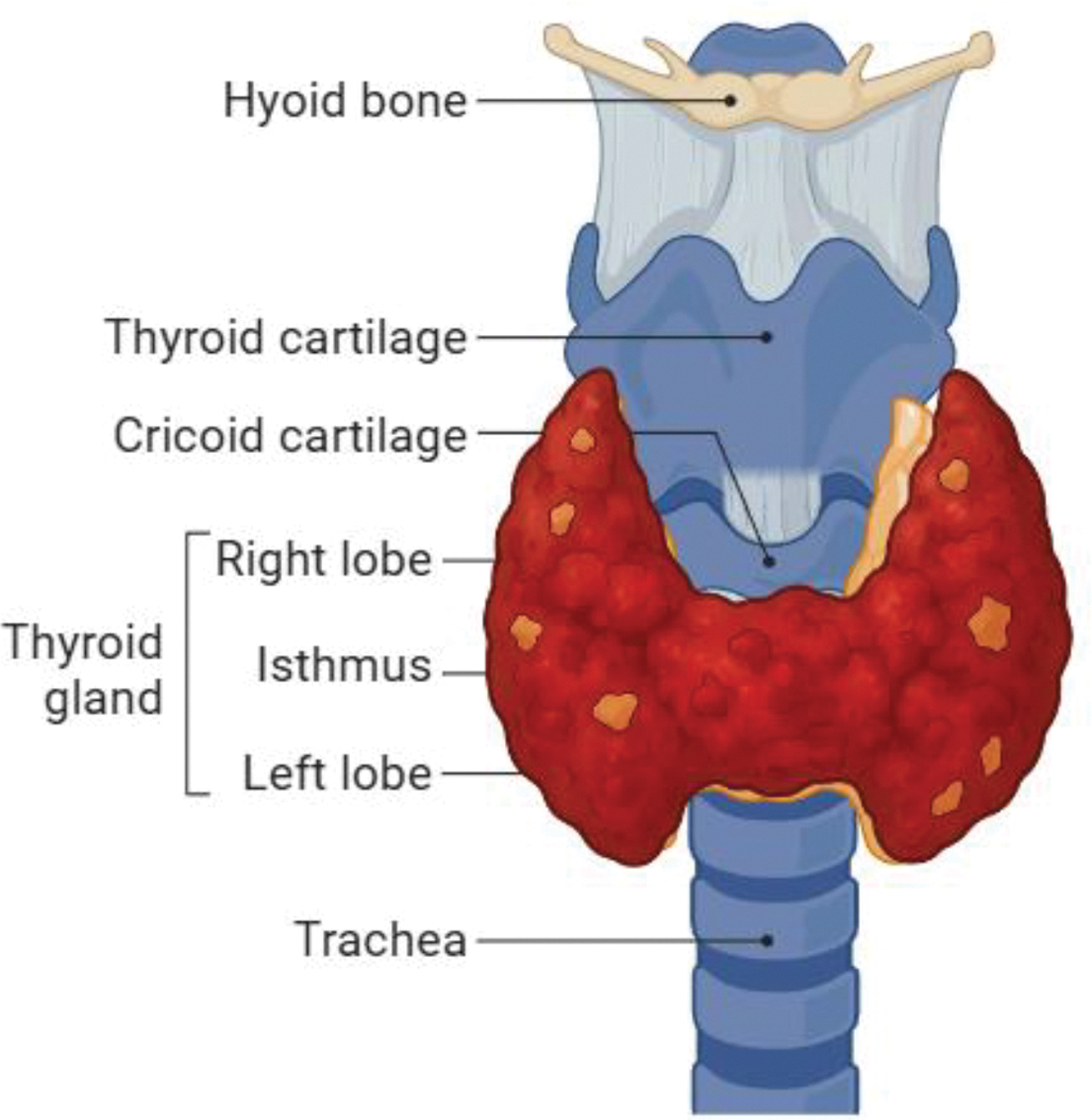
Anatomy of a thyroid affected by Hashimoto’s Thyroiditis. The thyroid gland is inflamed compared to a normal thyroid gland due to the destruction of thyrocytes.

The thyroid gland, located at the base of the neck, is a crucial endocrine organ responsible for synthesizing and secreting thyroid hormones: thyroxine (T4) and triiodothyronine (T3) (Figure 1). These hormones play a crucial role in regulating metabolism, growth, and development across various physiological systems. Structurally, the thyroid gland is composed of numerous follicles, which are spherical units lined by a single layer of follicular epithelial cells (thyrocytes) surrounding a central lumen filled with colloid [15,16]. Colloid primarily consists of thyroglobulin (Tg), a large glycoprotein that serves as the precursor for thyroid hormone synthesis and acts as a major thyroid autoantigen in HT [10]. Thyrocytes actively uptake iodide from the bloodstream via the sodium-iodide symporter (NIS), followed by its oxidation and incorporation into tyrosine residues on thyroglobulin, a process catalyzed by thyroid peroxidase (TPO) [15]. Both TPO and Tg are key autoantigens targeted by the immune system in HT [1,5,16]. The synthesis and release of thyroid hormones are tightly regulated by the hypothalamic-pituitary-thyroid (HPT) axis, primarily through the secretion of thyroid-stimulating hormone (TSH) by the pituitary gland. In HT, the chronic immune-mediated destruction of thyrocytes impairs the gland’s ability to produce sufficient hormones, leading to elevated TSH levels and clinical hypothyroidism [1,5].

## 2. Methodology

### 2.1 Search Strategy and Databases Utilized

This narrative review relied on a comprehensive search strategy across multiple electronic databases to identify relevant scientific literature on Hashimoto’s thyroiditis. The primary databases utilized included PubMed and Embase. Additional research was conducted on Scopus, BIOSIS, and Web of Science to ensure broad coverage.

The search terms were strategically combined to capture the breadth of the topic, encompassing aspects of the thyroid gland, the disease itself, and the associated immunological processes. Key phrases used in various combinations included: “Thyroid gland”, “Hashimoto’s thyroiditis”, “Immune system”, “Autoimmunity”, “Autoimmune thyroiditis”, “Lymphocytes”, “Antibodies”, “T-cells”, and “B-cells”. Other related terms, such as “Graves–Basedow disease” (for broader autoimmune thyroid disease context) and “vitamin D”, “cytokines”, and “anti-thyroid antibodies,” were also incorporated to identify specific mechanisms and interventions.

### 2.2 Inclusion and Exclusion Criteria

Articles were selected based on predefined inclusion and exclusion criteria to ensure the relevance and quality of the synthesized information. The primary inclusion criteria were: (i) Publication type: Original research articles, review articles, and meta-analyses, (ii) Language: English language publications only, (iii) Topic relevance: Studies directly investigating Hashimoto’s thyroiditis, its immunopathology, genetic and environmental factors, diagnostic approaches, and therapeutic interventions, (iv) Publication date: Although the primary focus was on research published between 2020 and 2025, seminal and foundational studies from earlier periods were also included to provide historical context and establish core concepts, (v) Study population: Both human and relevant animal studies were considered, particularly those elucidating molecular mechanisms transferable to human disease.

The exclusion criteria comprised: (i) Publication types: Case reports, editorials, and commentaries were generally excluded to maintain a focus on synthesized evidence and original research findings, and (ii) Irrelevance: Studies not directly about Hashimoto’s thyroiditis or its core immunological processes.

### 2.3 Data Extraction and Synthesis Approach

Following the identification of potentially relevant articles through systematic search, a multi-stage data extraction and synthesis process was employed. Initially, titles and abstracts were screened for relevance against the established inclusion criteria. Full-text articles of selected studies were then retrieved for detailed review. Key information extracted from each eligible article included: study design, participant characteristics, primary findings related to HT immunopathology, genetic and environmental associations, diagnostic methods, and treatment outcomes. Particular attention was given to data of specific immune cell subsets, cytokine profiles, autoantibody characteristics, and molecular pathways of thyroid cell damage. The synthesis approach for this narrative review involved a thematic analysis of the extracted data. Information was organized according to the key sections of the review, providing a comprehensive overview of the current understanding. Findings from different studies were compared to identify consistencies, discrepancies, and areas where further research is warranted. The aim was to integrate diverse findings into a coherent narrative, critically appraising the strengths and limitations of the existing evidence base, rather than performing a quantitative meta-analysis.

## 3. Risk Factors

Hashimoto’s thyroiditis arises from a complex interplay of genetic susceptibility and environmental influences [1,3,6]. While neither factor fully accounts for disease development, their cumulative effects are considered crucial in breaching immune tolerance and initiating thyroid destruction [3,8].

### 3.1 Genetic Predisposition

#### Familial Aggregation and Heritability:

Epidemiological studies consistently demonstrate a familial aggregation of HT and other autoimmune thyroid diseases (AITD), indicating a significant genetic component [3]. Heritability estimates for HT have been reported, emphasizing the complex nature of the genetic factors involved, including rare monogenic forms [3]. Twin studies further support a strong genetic influence, with higher concordance rates for HT in monozygotic twins compared to dizygotic twins [3]. This genetic susceptibility is not limited to thyroid-specific autoimmunity but often co-aggregates with other organ-specific autoimmune conditions, suggesting shared genetic pathways that predispose individuals to broader autoimmune diathesis [3,17].

#### HLA Typing and Susceptibility Genes:

The strongest genetic associations for HT reside within the major histocompatibility complex (MHC) genes, particularly human leukocyte antigen (HLA) class II alleles [1,3,5]. HLA-DR3 is one such allele frequently implicated in genetic predispositions [1,5]. Beyond HLA, numerous non-MHC genes involved in immune regulation have been identified as susceptibility loci [3]. These include genes encoding cytotoxic T-lymphocyte-associated protein 4 (CTLA-4), protein tyrosine phosphatase non-receptor type 22 (PTPN22), and forkhead transcription box protein P3 (FOXP3), all of which modulate T cell activation and regulatory T cell function [3,4,11]. Other genes, such as those related to thyroid-specific proteins (e.g., Thyroglobulin, TPO) and those associated with thyroid peroxidase antibody synthesis (e.g., BACH2), also contribute to genetic risk [3]. Variations in the SEPS1 gene, which encodes selenoprotein S, have also been linked to an increased risk of HT, particularly in male patients [18]. The cumulative effect of these genetic polymorphisms contributes to a breakdown in self-tolerance, facilitating the autoimmune response [1].

### 3.2 Environmental Triggers

Environmental factors are considered crucial additional contributors to HT development, often interacting with genetic predispositions [1,3,5].

#### Iodine Intake and Dietary Factors:

Iodine intake exhibits a complex relationship with HT prevalence, often described as a U-shaped curve where both deficiency and excess can influence disease risk [3]. Excessive iodine intake, particularly in genetically susceptible individuals, can increase the synthesis of highly iodinated thyroglobulin, potentially rendering it more immunogenic [3]. Other dietary factors and micronutrient deficiencies are also investigated for their role. Vitamin D deficiency is frequently observed in HT patients and has been correlated with a higher prevalence of thyroid autoimmunity [1,4,12,19–22]. Similarly, deficiencies in minerals such as zinc (Zn), selenium (Se), and magnesium (Mg) have been implicated, with selenium supplementation showing promise in reducing anti-thyroid antibody titers in some studies [1,5, 23–27]. Gluten-free diets have also been explored, with some evidence suggesting a reduction in thyroid antibody titers in patients with co-occurring celiac disease [28]. However, the evidence for a direct causal link for many of these dietary factors remains equivocal or requires further investigation [3,28].

#### Infections and Microbial Influences:

Infections are recognized as potential environmental triggers for autoimmunity, possibly operating through mechanisms like molecular mimicry [3,5]. While the direct link between specific infections and HT remains largely equivocal for many pathogens, congenital rubella and hepatitis C have been noted as potential contributors [3]. The gut microbiota is also emerging as an important environmental modulator of immune responses, and intestinal dysbiosis has been suggested as a factor in HT pathogenesis [3,5]. Chronic stress, smoking, alcohol consumption, and certain medications (e.g., lithium, immune checkpoint inhibitors) are additional environmental elements that may influence HT risk or progression [3,5,29].

### 3.3 Hormonal and Gender-Related Factors

Hormonal influences and gender disparities are well-established epidemiological features of HT, with women exhibiting a significantly higher incidence than men [3].

#### Estrogen Effects and Sex Differences:

The disproportionate prevalence of HT in females suggests a substantial role for sex hormones, particularly estrogens, in modulating immune responses [3,30,31]. Estrogens are known to have immunomodulatory effects, often enhancing humoral immunity and potentially contributing to the predisposition to autoimmune diseases in women [3]. The age of onset for HT commonly aligns with periods of significant hormonal fluctuation, such as puberty, pregnancy, and menopause, further supporting a hormonal influence [3,31–33].

#### Pregnancy and Postpartum Considerations:

Pregnancy represents a unique physiological state with profound immunological and hormonal shifts, impacting the course of autoimmune diseases [3,5]. While pregnancy itself often leads to a temporary remission or stabilization of some autoimmune conditions due to a shift towards a Th2-dominant immune response, the postpartum period is frequently associated with exacerbation or onset of autoimmune thyroiditis [3,34]. This phenomenon, known as postpartum thyroiditis, shares immunopathological features with HT and is characterized by a temporary autoimmune attack on the thyroid, sometimes leading to permanent hypothyroidism [34]. The rebound in immune activity following the withdrawal of pregnancy-related immunosuppression is considered a key factor [3,34].

## 4. Cellular and Molecular Mechanisms of Hashimoto’s Thyroiditis

The pathogenesis of Hashimoto’s thyroiditis is orchestrated by a complex array of cellular and molecular immune events that culminate in the destruction of thyroid follicular cells [1,8,35]. The core of the autoimmune process in HT is a breakdown in self-tolerance to thyroid autoantigens [1].

### 4.1 Lymphocytic Infiltration of the Thyroid

A hallmark pathological feature of HT is the extensive lymphocytic infiltration within the thyroid parenchyma [1]. This infiltrate comprises various immune cell populations, including small lymphocytes, plasma cells, macrophages, and often well-developed germinal centers [1,5]. The presence of these immune cells directly contributes to the inflammatory and destructive processes [5].

### 4.2 T-Cell Subsets and Their Roles

**Figure 2:**
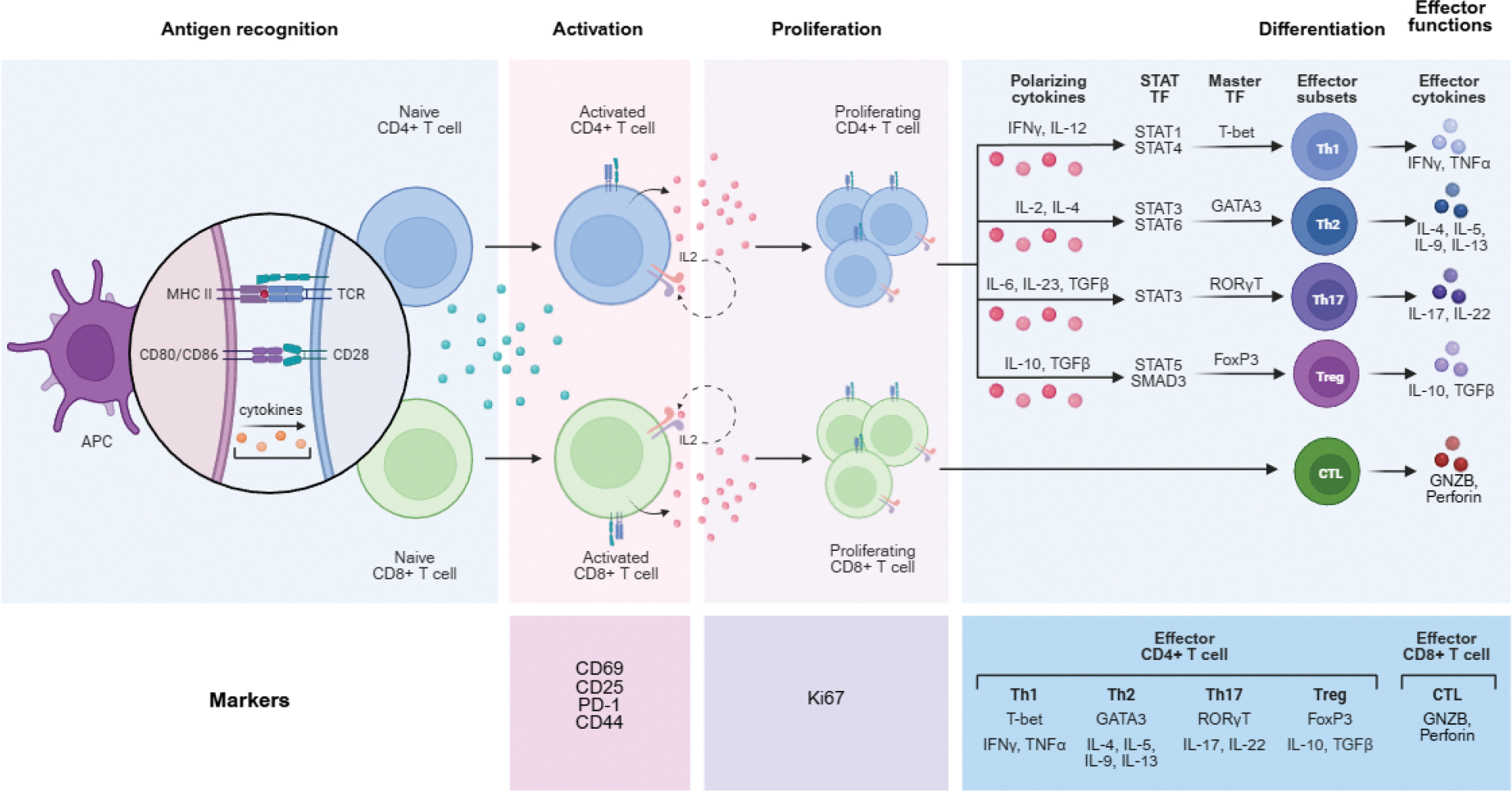
T cell Types and Effective Cytokines. T-cell activation from antigen recognition, their proliferation, and their differentiation into effector subsets and effector cytokines.

T lymphocytes, particularly CD4+ T helper cells and CD8+ cytotoxic T lymphocytes (CTLs), play central roles in HT immunopathology [1,5,36]. The CD4:CD8 ratio in the thyroid infiltrate in HT is typically elevated, often around 4:1, suggesting a prominent role for CD4+ T cells (Figure 2) [1,36].

#### Th1 Cells:

**Figure 3:**
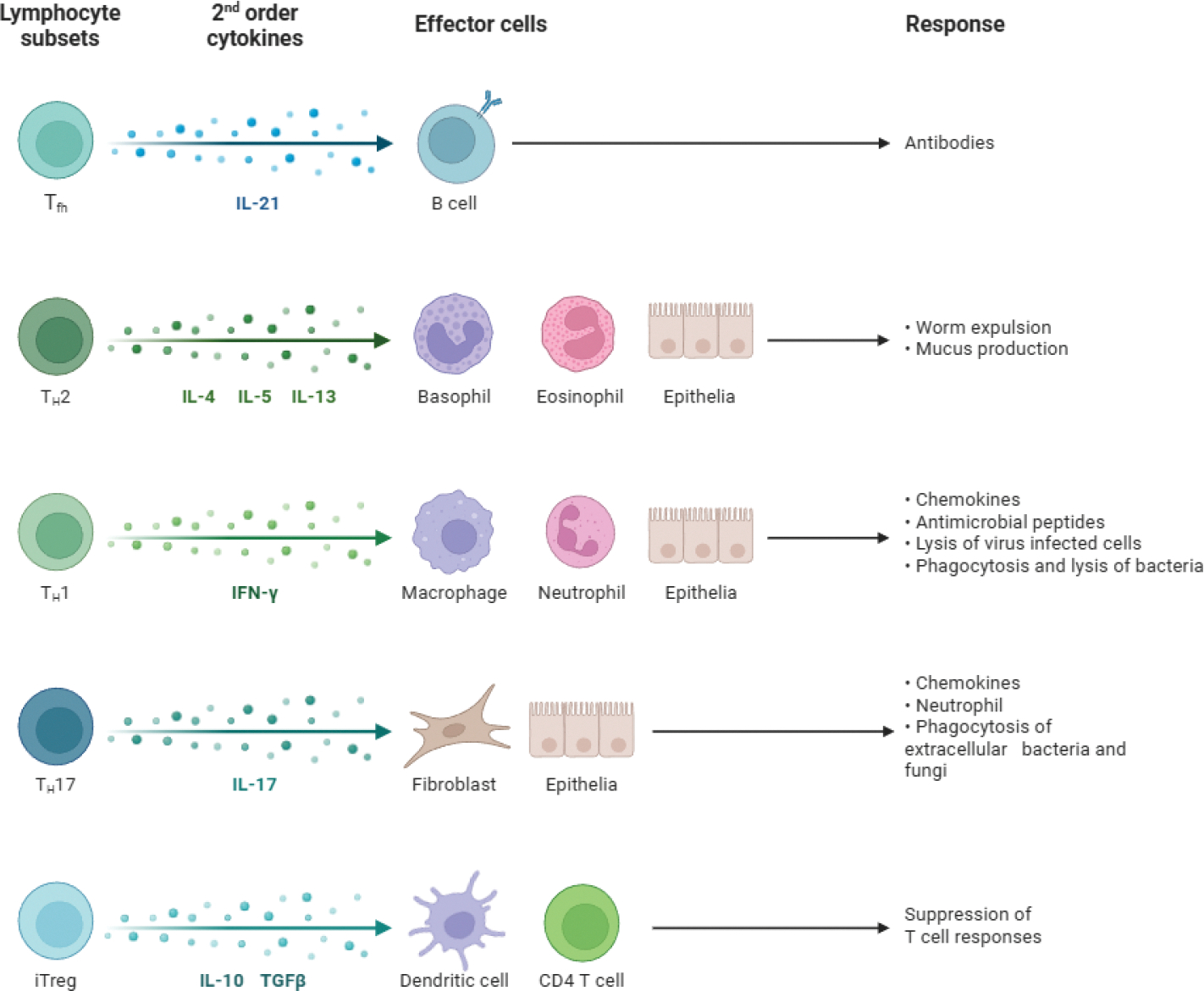
Effector Responses Induced by 2nd Order Cytokines. The cytokines that are altered in patients with HT include IL-12 & IFN-γ, which produce the proinflammatory cytokines that destroy the thyrocytes.

Historically, HT has been considered a Th1-dominant disease (Wronska et al., 2024; other). Th1 lymphocytes, upon stimulation by IL-12 and IFN-γ, differentiate from naive CD4+ T cells [1,5]. They produce proinflammatory cytokines, notably IFN-γ, which directly damages thyrocytes by inducing FAS expression on their surface, and also activate macrophages and CD8+ T cells (Figure 3) [1,5]. While some studies suggest a relative decrease in peripheral Th1 cells in HT, an increased immune deviation of Th1 lymphocytes correlates with the progression towards overt hypothyroidism (hypothyroid).

#### Th17 Cells:

Recent research indicates a significant contribution of Th17 lymphocytes to the pathogenesis of HT [1,36–38]. These cells promote inflammation and secrete cytokines, including IL-17, IL-21, and IL-22 [1,36–38]. IL-17 exacerbates inflammation, while IL-22 levels are significantly increased in newly diagnosed, untreated patients with HT [5]. An imbalance between Th17 and regulatory T cells (Tregs) is considered central to the disease [5].

#### Treg Cells:

Regulatory T cells (Tregs) play a crucial role in maintaining immune tolerance [5]. Defects in Treg function or reduced numbers are implicated in the loss of self-tolerance in HT [3]. While some studies suggest an increased proportion of peripheral Tregs in HT as a compensatory mechanism, an imbalance in the Th17/Treg ratio is often observed [37,38].

#### CD8+ T Cells:

Cytotoxic T lymphocytes (CTLs) directly destroy thyroid cells through mechanisms involving perforin and granzymes or the Fas-FasL cascade [1,5]. Both TPO and Tg are recognized by CD8+ T cells, which are involved in the destruction of the thyroid [39].

### 4.3 B-Cell Activation and Autoantibody Production

B lymphocytes, often found within the thyroid’s germinal centers, are activated by T follicular helper (Tfh) cells and contribute significantly to HT pathogenesis through the production of autoantibodies [1,5,12]. The primary autoantibodies detected in HT are anti-thyroid peroxidase (TPOAb) and anti-thyroglobulin (TgAb) [1,5]. TPOAb is a clinical marker that confirms the presence of the disease [5]. These antibodies contribute to thyrocyte destruction through antibody-dependent cell-mediated cytotoxicity (ADCC) [12,40]. The N-glycosylation of the Fc fragment of IgG can influence its effector functions, with changes in glycosylation enhancing cytotoxicity in HT [40]. While less common, TSH receptor-stimulating antibodies (TSAb), typically associated with Graves’ disease, can also be found in a subset of HT patients, particularly those with thyroid-associated orbitopathy [41].

### 4.4 Antigen Presentation and Loss of Immune Tolerance

The initiation of the autoimmune cascade in HT fundamentally involves the aberrant presentation of thyroid autoantigens and the subsequent breakdown of immune tolerance [5].

### 4.5 Dendritic Cell Functionality

Antigen-presenting cells (APCs), primarily dendritic cells (DCs) and macrophages, play a crucial role as early participants in HT [1,5]. These cells accumulate in the thyroid during the initial stages of autoimmunization and express MHC class II molecules [5]. They present organ-specific autoantigens to naive CD4+ T cells, thereby activating them and initiating their differentiation into various T helper subsets [1,5]. Interestingly, thyrocytes themselves can be induced to express MHC class II molecules, particularly under the influence of cytokines like IFN-γ, enabling them to act as non-professional APCs and further contribute to T cell activation and the perpetuation of the autoimmune response [5].

### 4.6 Molecular Mimicry Hypotheses

The concept of molecular mimicry provides a theoretical framework for understanding how environmental factors, such as infections, may trigger autoimmunity [3]. This hypothesis suggests that microbial antigens may exhibit structural similarities with self-antigens present in the thyroid [3]. An immune response mounted against the foreign pathogen could then inadvertently cross-react with thyroid autoantigens, leading to the initiation or exacerbation of HT [3]. While specific microbial agents have been investigated, definitive evidence for molecular mimicry as a primary trigger in human HT remains an active area of research [3].

### 4.7 Cytokine Networks and Inflammatory Mediators

Cytokines, small signaling proteins, form intricate networks that regulate the immune response and contribute to the inflammatory environment in the thyroid gland during HT [3,5].

#### Proinflammatory versus Regulatory Cytokines:

An imbalance between proinflammatory and anti-inflammatory cytokines is characteristic of HT [5]. Proinflammatory cytokines, such as IL-2, IL-12, IL-17, IL-21, IL-22, IFN-γ, and TNF-α, are often elevated [5,42,43]. IFN-γ, primarily from Th1 cells, directly damages thyroid cells and promotes MHC class II expression on thyrocytes (Figure 3) [1,5]. IL-17, secreted by Th17 cells, exacerbates inflammation [5,36–38]. IL-23, produced by non-specific immune cells, may also influence the intensity of the Th1 response and is found elevated in some HT patients [5]. The inflammasome, a multiprotein complex, can be activated by IL-1β and IL-18, triggering pyroptosis, a form of inflammatory cell death in HT [5]. Conversely, anti-inflammatory cytokines, such as IL-10 and IL-2, are often reduced, contributing to the persistent inflammatory state [5].

#### Chemokine Signaling in Thyroid Tissue:

Chemokines are small cytokines that mediate cell chemotaxis, guiding immune cells to sites of inflammation [1]. In HT, the release of proinflammatory cytokines and chemokines by damaged thyrocytes and infiltrating immune cells creates a feedback loop that sustains and amplifies the immune process [1]. Specific chemokines, such as CXCL10, are produced by thyrocytes and contribute to the recruitment of T cells into the thyroid gland, further fueling the inflammatory infiltrate [1]. The persistent presence of these chemokines helps maintain the characteristic lymphocytic infiltration observed in HT [1].

### 4.8 Apoptosis and Thyrocyte Destruction

The outcome of the sustained immune attack in HT is the destruction of thyroid follicular cells (thyrocytes), leading to reduced hormone production and hypothyroidism [1,5,12].

#### Molecular Pathways of Cell Death:

Thyrocyte destruction in HT primarily occurs through programmed cell death mechanisms, notably apoptosis [5,12]. Key pathways involved include the Fas-FasL cascade and the action of perforin and granzymes [1]. CD8+ cytotoxic T cells induce apoptosis by releasing perforin, which forms pores in the target cell membrane, allowing granzymes to enter and activate downstream caspases, leading to cell death [1,5]. Additionally, the binding of Fas ligand (FasL) on activated immune cells to Fas receptors on thyrocytes triggers apoptotic signaling pathways [1]. Antibody-dependent cell-mediated cytotoxicity (ADCC), mediated by anti-TPO and anti-Tg antibodies, also contributes to the apoptosis of thyrocytes [12]

#### Oxidative Stress and Cellular Injury:

Oxidative stress is an additional factor contributing to cellular injury and thyrocyte destruction in HT [44]. Studies indicate that oxidative stress increases progressively through the euthyroid, subclinical hypothyroid, and overt hypothyroid stages of HT [44]. Elevated levels of total oxidant status (TOS) and oxidative stress index (OSI), alongside reduced total antioxidant status (TAS), are observed in HT patients, particularly as hypothyroidism develops [44]. The production of reactive oxygen species (ROS) by infiltrating immune cells and thyrocytes themselves can induce cellular damage and contribute to the apoptotic process [44]. For instance, IL-23 has been shown to induce ROS accumulation and suppress autophagy in thyroid follicular cells from patients with HT, highlighting a potential pathway for cellular injury.

## 5. Diagnosis

Accurate diagnosis of Hashimoto’s thyroiditis relies on a combination of clinical assessment, laboratory evaluation, and, in some cases, imaging studies. These modalities collectively provide a comprehensive picture of thyroid function and the underlying autoimmune process.

### 5.1 Clinical Presentation and Symptomatology

The clinical presentation of HT can be highly variable, ranging from asymptomatic euthyroidism to overt hypothyroidism [45–47]. Initially, patients may experience symptoms related to a fluctuating thyroid state, or mild symptoms of hypothyroidism such as fatigue, weight gain, cold intolerance, constipation, and dry skin [29,45]. The thyroid gland may be enlarged (goiter), which can be either diffuse or nodular, and may occasionally be tender [29,45]. In some instances, transient hyperthyroidism, known as hashitoxicosis, may occur due to the release of preformed thyroid hormones from damaged follicular cells [15,29,45]. As the disease progresses, permanent hypothyroidism typically develops [1]. Rarely, severe neurological complications such as Hashimoto’s encephalopathy, characterized by cognitive impairment, seizures, or psychiatric symptoms, can occur, often responsive to steroid treatment even with a standard thyroid profile [45–48]. Other autoimmune comorbidities, such as pernicious anemia or celiac disease, may also present concurrently [49,50].

### 5.2 Laboratory Evaluation of Thyroid Function and Antibodies

Laboratory tests are indispensable for confirming the diagnosis of HT and assessing thyroid functional status.

#### Thyroid Peroxidase and Thyroglobulin Antibodies:

The measurement of anti-thyroid peroxidase antibodies (TPOAb) and anti-thyroglobulin antibodies (TgAb) serves as the primary immunological diagnostic marker for HT [1,5]. Elevated titers of TPOAb are particularly sensitive and specific for HT, often present even before overt thyroid dysfunction manifests [1,5]. TgAb detection supplements TPOAb, providing additional diagnostic support, though it is generally less specific than TPOAb2 [51]. The presence of these autoantibodies indicates an ongoing autoimmune attack on the thyroid gland [1,5].

#### Thyroid-Stimulating Hormone and Hormonal Profiles:

Assessment of thyroid-stimulating hormone (TSH), free thyroxine (fT4), and free triiodothyronine (fT3) levels is crucial for evaluating thyroid function in HT [1,52].

#### Euthyroidism:

In the early stages of HT, patients may be euthyroid (normal TSH, fT4, fT3) despite the presence of autoantibodies [46,47].

#### Subclinical Hypothyroidism:

As thyroid damage progresses, TSH levels may rise above the normal range, while free T4 (fT4) and free T3 (fT3) levels remain within normal limits. This state, known as subclinical hypothyroidism, often precedes overt hypothyroidism [53].

#### Overt Hypothyroidism:

With continued destruction of thyrocytes, TSH levels become markedly elevated, and fT4 and fT3 levels fall below the normal range, indicating overt hypothyroidism [1]. This typically necessitates thyroid hormone replacement therapy [1,53].

### 5.3 Imaging Modalities in Diagnosis

Imaging techniques provide valuable insights into the structural changes within the thyroid gland, complementing serological and functional assessments.

#### Ultrasonography Findings:

Thyroid ultrasonography (USG) is a highly useful tool in the diagnosis and monitoring of HT, particularly in patients where autoantibodies may not be detected or in cases of equivocal serology [5]. Characteristic USG findings suggestive of HT include decreased echogenicity (hypoechogenicity), heterogeneity of thyroid parenchyma, excessive vascularity, and the presence of small cysts [5]. These features reflect the ongoing lymphocytic infiltration, inflammation, and fibrotic changes within the gland [5]. USG can also identify thyroid nodules, which are common in HT, and guide fine-needle aspiration (FNA) if malignancy is suspected [54].

#### Other Radiological Techniques:

While ultrasonography is typically sufficient for thyroid imaging in HT, other radiological techniques may be employed in specific clinical scenarios. Computed tomography (CT) or magnetic resonance imaging (MRI) of the neck may be used to evaluate the extent of goiter, assess compression symptoms, or investigate suspected thyroid lymphoma, a rare complication of chronic HT [55,56]. Radioiodine uptake scans are typically not used for routine diagnosis of HT but can help differentiate HT from other causes of thyroid dysfunction, such as Graves’ disease or subacute thyroiditis [15].

## 6. Current Treatments

The management of Hashimoto’s thyroiditis primarily focuses on alleviating symptoms of hypothyroidism and preventing its progression. Current therapeutic approaches range from conventional pharmacological treatments to adjunctive therapies and lifestyle modifications.

### 6.1 Conventional Pharmacological Management

#### Levothyroxine Replacement Therapy:

Levothyroxine (L-T4) replacement therapy constitutes the cornerstone of conventional pharmacological management for HT patients who develop hypothyroidism 1. This synthetic form of T4 effectively restores euthyroid, alleviates hypothyroid symptoms, and normalizes elevated TSH levels [1,53,57,58]. L-T4 treatment is generally initiated when TSH levels are consistently elevated or when clinical symptoms of hypothyroidism are present, even in the setting of subclinical hypothyroidism [1]. In some pediatric cases, L-T4 therapy has been shown to reduce thyroid volume; however, its effect on thyroid function and antibody levels may be limited to specific time periods [53,57]. Normalizing TSH levels with L-T4 may also reduce the autoantigenic properties of thyrocytes, potentially contributing to a decrease in autoantibody titers [53,57].

#### Dose Adjustment Paradigms:

The appropriate dosage of levothyroxine is individually tailored to achieve and maintain TSH levels within the target reference range, which typically ranges from 0.4 to 4.0 mIU/L; however, individual patient factors may warrant different targets. Dose adjustments are commonly based on regular monitoring of TSH and free T4 levels, typically every 6–8 weeks until the dose is stable, and then annually or as clinically indicated. Factors influencing L-T4 dosage include body weight, age, comorbidities, concomitant medications (e.g., iron, calcium, and proton pump inhibitors that can impair absorption), and pregnancy status [59]. Adherence to therapy is critical for optimal outcomes, as HT often necessitates lifelong treatment [1,53,57].

### 6.2 Novel and Adjunctive Treatments

Beyond standard L-T4 replacement, various novel and adjunctive treatments are being explored to address the autoimmune component of HT or improve patient outcomes.

#### Immunomodulatory Agents:

Given the autoimmune nature of HT, immunomodulatory agents have been investigated, though their widespread clinical application is limited. Glucocorticoids, for instance, are primarily used for acute, severe complications like Hashimoto’s encephalopathy, where they often elicit a strong response [60,61]. Other agents, such as statins, have been shown to reduce thyroid autoimmunity, with high-dose statin therapy producing a more substantial effect on TPOAb and TgAb titers compared to combination therapies [62]. However, these treatments are not routinely employed for HT due to concerns about side effects and the availability of effective L-T4 therapy.

### 6.3 Lifestyle Modifications and Dietary Interventions

Lifestyle and dietary interventions are increasingly recognized as potential adjunctive strategies to manage HT, particularly concerning modifiable risk factors [1].

#### Vitamin D Supplementation:

**Figure 4:**
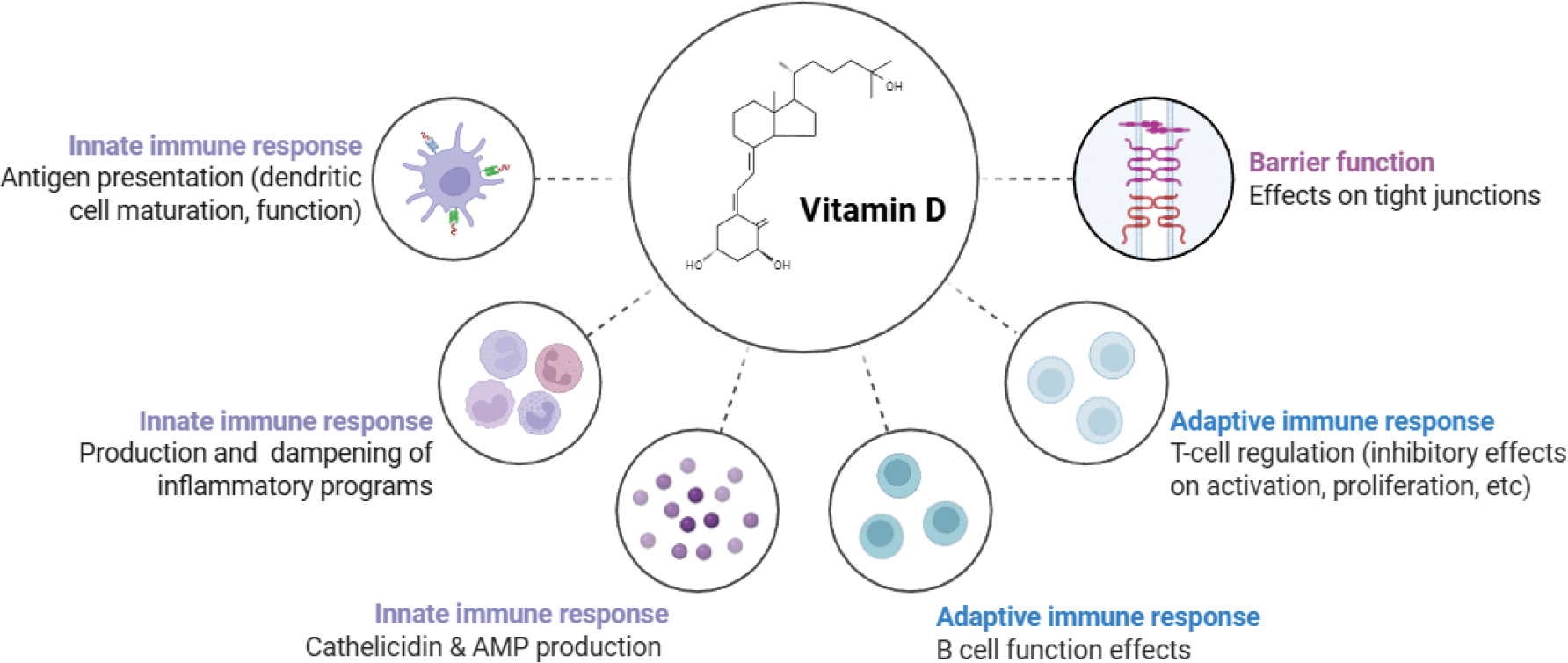
Effects of Vitamin D on the Immune System. Vitamin D plays a crucial role in both innate and adaptive immunity by acting as an immunomodulator by regulating immune cell activity. Vitamin D supplementation is common in patients with HT.

Vitamin D deficiency is common in HT patients [1,4,12,19–22]. Supplementation with vitamin D has shown promise in reducing antithyroid antibody levels, improving thyroid function, and modulating immune markers, such as cytokines and T-cell subsets (Figure 4) [1,4,12,19–22].

#### Selenium Supplementation:

Selenium plays a crucial role in thyroid metabolism and helps protect against oxidative damage [23–27]. Supplementation has been observed to reduce TPOAb titers and inflammatory markers in some HT patients, particularly when co-administered with levothyroxine [1,23–27].

#### Dietary Changes:

Beyond specific micronutrients, broader dietary patterns have been explored. For instance, a gluten-free diet has shown some benefit in reducing thyroid antibody titers in HT patients with incidentally found positive anti-tissue transglutaminase antibodies, suggesting a possible role in those with celiac disease or gluten sensitivity [28,63].

#### Stress Management:

Chronic stress is an environmental factor implicated in HT [5]. Stress management techniques are considered part of a holistic approach to managing autoimmune conditions [1,64].

Anatabine, an alkaloid found in tobacco plants, has demonstrated an immunological effect on TgAb levels, resulting in a significant reduction in absolute serum TgAb in HT patients [65]. This finding warrants further investigation into its long-term effects [65].

### 6.4 Treatment of Comorbidities and Complications

Effective management of HT also involves addressing associated comorbidities and potential complications. HT frequently co-occurs with other autoimmune conditions, such as celiac disease, pernicious anemia, or type 1 diabetes [49,50]. Screening for and managing these conditions is crucial for comprehensive patient care. Complications like thyroid-associated orbitopathy, though rare in HT, may require specific interventions if TSH receptor antibodies are present [41]. The rare but severe Hashimoto’s encephalopathy necessitates prompt diagnosis and aggressive steroid therapy [60,61]. Furthermore, the relationship between HT and thyroid cancer, particularly papillary thyroid cancer (PTC), requires careful consideration, as HT can positively influence the prognosis of intrathyroidal PTC [66–68].

## 7. Conclusions

Literature provides a robust foundation for understanding Hashimoto’s thyroiditis as a multifactorial autoimmune disorder. A synthesis of key findings across mechanistic, diagnostic, and therapeutic domains reveals areas of established knowledge and persistent challenges.

Mechanistic studies consistently establish HT as a T-cell-mediated autoimmune disease, with significant contributions from both humoral and cellular immunity [1,5,8]. Initial views emphasizing a predominant Th1 response are now augmented by evidence for the significant involvement of Th17 cells and an imbalance in the Th17/Treg axis [5,36–38]. The role of CD8+ T cells in direct thyrocyte cytotoxicity is also well-supported [5,42]. The production of TPOAb and TgAb by B cells, facilitated by T follicular helper cells, and their involvement in ADCC pathways, underscores the humoral component [5,12]. Genetic predispositions, particularly HLA and immunoregulatory genes like CTLA-4 and PTPN22, are consistently identified across studies as foundational to susceptibility [3,5]. Environmental triggers, including iodine intake, infections, and micronutrient deficiencies (e.g., vitamin D, selenium), are recurrently discussed, albeit with varying degrees of conclusive evidence for direct causation across literature [1,3,5]. The concept of cumulative weaknesses, akin to a ‘Swiss cheese’ model, aptly captures the multifactorial etiology [69].

Diagnostic practices for HT are well-established, combining clinical evaluation, serological testing, and imaging [70]. The detection of TPOAb remains the most sensitive and specific serological marker, often preceding the onset of clinical hypothyroidism [1,5]. TSH and free thyroid hormone measurements reliably assess functional status and guide treatment initiation. Thyroid ultrasonography is a valuable non-invasive tool, offering characteristic findings (hypoechogenicity, heterogeneity, and increased vascularity) that support diagnosis, especially in antibody-negative cases or for evaluating nodules [5].

## 8. Discussion

Despite significant advancements in understanding Hashimoto’s thyroiditis, several challenges and unresolved issues persist, hindering optimal patient management and the development of curative therapies.

### 8.1 Controversies in Pathogenesis and Progression of Hashimoto’s Thyroiditis Disease

While the general framework of HT pathogenesis is established, the exact molecular mechanisms by which immune dysfunction leads to thyroid tissue destruction remain incompletely explained [8]. The precise triggers that initiate the autoimmune cascade in genetically susceptible individuals are often elusive, making prevention difficult. Controversies exist regarding the relative importance of various environmental factors and their interaction with specific genetic variants. Furthermore, the progression of HT is highly variable, with some patients maintaining euthyroidism for years while others rapidly develop overt hypothyroidism. The factors influencing this differential progression are not fully understood. The frequent coexistence of HT with other autoimmune diseases, such as multiple sclerosis, celiac disease, and primary biliary cholangitis, suggests shared underlying mechanisms, yet the exact nature of these common pathways requires further elucidation [49,50]. The rare, but serious, complication of Hashimoto’s encephalopathy also highlights an area where the link between thyroid autoimmunity and neurological dysfunction is not fully characterized [46–48].

### 8.2 Limitations of Diagnostic Criteria and Biomarkers

Current diagnostic criteria, relying on TSH levels, free thyroid hormones, and anti-TPO/anti-Tg antibodies, are generally effective, but limitations exist. A notable challenge involves the 5–10% of HT patients who do not present with detectable anti-thyroid antibodies, making diagnosis more complex and reliant on imaging or biopsy [5]. Furthermore, the clinical significance of macro-TSH, which can lead to diagnostical and therapeutical difficulties due to anti-TSH autoantibodies, presents a diagnostic pitfall that requires careful consideration [71]. Biomarkers predicting disease progression, treatment response, or the development of complications remain largely insufficient. Improved biomarkers are necessary for identifying individuals at high risk, monitoring the effectiveness of immune-modulating therapies, and distinguishing HT from other thyroid conditions with similar presentations.

### 8.3 Therapeutic Gaps and Unmet Clinical Needs

A significant therapeutic gap in HT is the absence of an effective treatment that directly targets and resolves the underlying autoimmunity [52,69]. While levothyroxine therapy effectively manages hypothyroidism, many patients continue to experience persistent symptoms, such as fatigue, cognitive impairment, and musculoskeletal pain, despite achieving biochemical euthyroid [11]. This highlights an unmet clinical need for interventions that address autoimmune inflammation and its extrathyroidal effects. The role and optimal dosage of nutritional interventions, such as selenium and vitamin D supplementation, require further robust clinical trials to establish definitive guidelines [72]. Moreover, personalized treatment strategies that consider individual genetic profiles, immune phenotypes, and environmental exposures are yet to be fully developed and integrated into clinical practice.
